# The host niches of soybean rather than genetic modification or glyphosate application drive the assembly of root‐associated microbial communities

**DOI:** 10.1111/1751-7915.14164

**Published:** 2022-11-06

**Authors:** Minkai Yang, Fuhe Luo, Yuchen Song, Shenglin Ma, Yudi Ma, Aliya Fazal, Tongming Yin, Guihua Lu, Shucun Sun, Jinliang Qi, Zhongling Wen, Yongchun Li, Yonghua Yang

**Affiliations:** ^1^ Institute for Plant Molecular Biology, State Key Laboratory of Pharmaceutical Biotechnology, School of Life Sciences Nanjing University Nanjing China; ^2^ Co‐Innovation Center for Sustainable Forestry in Southern China Nanjing Forestry University Nanjing China; ^3^ School of Life Sciences Huaiyin Normal University Huaian China; ^4^ State Key Laboratory of Subtropical Silviculture, College of Environmental and Resource Sciences Zhejiang A&F University Hangzhou China

## Abstract

Plant roots significantly influence soil microbial diversity, and soil microorganisms play significant roles in both natural and agricultural ecosystems. Although the genetically modified (GM) crops with enhanced insect and herbicide resistance are thought to have unmatched yield and stress resistance advantages, thorough and in‐depth case studies still need to be carried out in a real‐world setting due to the potential effects of GM plants on soil microbial communities. In this study, three treatments were used: a recipient soybean variety Jack, a triple transgenic soybean line JD321, and the glyphosate‐treated JD321 (JD321G). Three sampling stages (flowering, seed filling and maturing), as well as three host niches of soybean rhizosphere [intact roots (RT), rhizospheric soil (RS) and surrounding soil (SS)] were established. In comparison to Jack, the rhizospheric soil of JD321G had higher urease activity and lower nitrite reductase at the flowering stage. Different treatments and different sampling stages existed no significant effects on the compositions of microbial communities at different taxonomic levels. However, at the genus level, the relative abundance of three plant growth‐promoting fungal genera (i.e. *Mortierella*, *Chaetomium* and *Pseudombrophila*) increased while endophytic bacteria *Chryseobacterium* and pathogenic bacteria *Streptomyces* decreased from the inside to the outside of the roots (i.e. RT → RS → SS). Moreover, two bacterial genera, *Bradyrhizobium* and *Ensifer* were more abundant in RT than in RS and SS, as well as three species, *Agrobacterium radiobacter*, *Ensifer fredii* and *Ensifer meliloti*, which are closely related to nitrogen‐fixation. Furthermore, five clusters of orthologous groups (COGs) associated to nitrogen‐fixation genes were higher in RT than in RS, whereas only one COG annotated as dinitrogenase iron‐molybdenum cofactor biosynthesis protein was lower. Overall, the results imply that the rhizosphere host niches throughout the soil–plant continuum largely control the composition and function of the root‐associated microbiome of triple transgenic soybean.

## Graphical abstract

The host niche rather than newly introduced genes and glyphosate application determines the differentiation of root‐associated microbial communities. The growth stages of soybean affect the nutrient dynamics and microbial communities. GM soybean with glyphosate application only altered the rhizosphere nutrient dynamics, rather than the assembly and shift of microbial communities.

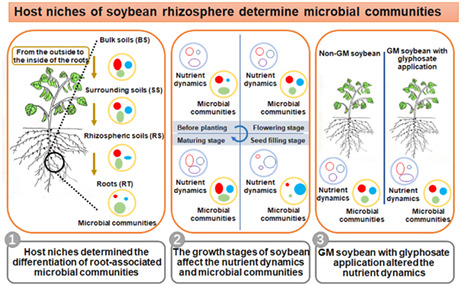

## INTRODUCTION

The safety of genetically modified (GM) crops is one of the hot issues around the world. According to the report of the International Service for the Acquisition of Agri‐biotech Applications (ISAAA), the planting area of GM crops has increased from 1.7 million hectares to 190.4 million hectares in the past two decades, since GM crops provide food, feed and shelter for the 7.7 billion global population (ISAAA, 2019). Unfortunately, despite the incomparable advantages of GM crops, since their introduction, widespread applications have sparked persistent public concerns across the globe. Evaluating the possible negative effects of alien genes expressed in transgenic plants is critical in determining environmental safety. Microbial community analysis of the rhizosphere is a vital approach for evaluating the environmental impact of GM crops (Oh et al., 2021).

Many prior studies have revealed the varied impacts of GM crops on the environment, particularly on the root‐associated microbial communities (Dunfield & Germida, 2004; Guan et al., 2016; Liu et al., 2005; Wen et al., 2019; Yang et al., 2020). The two most common target genes for GM crops are the 5‐enolpyruvylshikimate‐3‐phosphate synthase (*EPSPS*) gene and *cry1c* gene. The *EPSPS* gene can improve glyphosate tolerance, while the *cry1c* gene can increase insect resistance (de Maagd et al., 2001; Duke & Powles, 2008; Guo et al., 2016; Yang et al., 2020). According to several reports, glyphosate‐tolerant transgenic crops may have a significant impact on the rhizosphere's microbial communities, particularly by reducing diversity and influencing the composition of the culturable root‐endophytic bacterial community (Dunfield & Germida, 2001; Lee et al., 2011; Lopes et al., 2016). On the contrary, certain other *EPSPS*‐transgenic crops had little to no impact on the overall bacterial community in the rhizosphere (Wen et al., 2019; Yang et al., 2021, 2020). For insect‐resistance transgenic crops, the adsorption and binding of Bt toxin proteins to clay could result in toxin accumulation and may further affect soil microbes (Li et al., 2018; Strain & Lydy, 2015). In previous studies, Bt crops were found to dramatically impact the microbial properties and enzymatic activities in rhizosphere soil, as well as to reduce the gram‐positive to gram‐negative bacteria ratio in rhizosphere soil (Chen et al., 2012; Xue et al., 2005). In contrast, some Bt crops were shown to have no impact on the soil archaeal community or enzyme activity when compared to their parental crops (Coz et al., 2008; Wang, Wang, et al., 2017). The inconsistent effects of GM crops on the rhizosphere community could possibly be attributed to soil heterogeneity and a lack of effective control (Mandal et al., 2020). In fact, there is also a lack of evaluation of the practical effects of GM crops with insect‐resistance and glyphosate‐tolerance on rhizosphere microorganisms and nutrient dynamics under glyphosate application.

Glyphosate has been well known for being environmentally friendly and toxicologically safe (Duke & Powles, 2008). However, the potential effects of glyphosate on plant mineral nutrition, diseases and soil microorganisms have attracted increasing attention (Duke & Powles, 2008; Johal & Huber, 2009; Sihtmäe et al., 2013). Through the analysis of more than 8000 citations, Duke et al. (2012) believed that whether the glyphosate application has a significant impact on mineral nutrition on crops is still controversial. The impact of glyphosate on soil microbiome may be masked by the redundant function of microbial community, and glyphosate treatment would further alter the composition of soil microbial community and influence several critical processes mediated by particular microbial groups (Imfeld & Vuilleumier, 2012).

Plant host can make primary resources available, such as sugars and other chemicals, via root exudates, and hence strongly select specific microbial groups surrounding the roots (Kroll et al., 2017). Different crop microbiomes were dominated by different dominant taxa (e.g. Bacilli for wheat and barley and Methylobacteriaceae for maize) (Xiong et al., 2020). Soybean is the most widely grown oil‐bearing crop in the world, accounting for more than 50% of the world's edible oil production, and GM soybean occupies about 74% of the soybeans planted in the world (ISAAA, 2019). Some plant growth‐promoting rhizobacteria (PGPR) such as *Rhizobium*, *Novosphingobium* and *Streptomyces* have been found to be abundant in soybean rhizosphere (Liu et al., 2019). A recent study also demonstrated that the compartment niche and the host species have a major influence on the microbiome assembly along the soil–plant continuum as a result of the enhanced host selection pressure from soils to epiphytes to endophytes (Xiong et al., 2020). Although triple transgenic insect‐resistant and glyphosate‐tolerant soybeans offer more promising and superior agronomic and resistance traits, their impacts on the composition and function of root‐associated microbiomes in rhizosphere host niches throughout the soil–plant continuum remain unknown.

To address these issues, a triple transgenic (*g2m‐epsps* & *gr79m‐epsps* & *cry1c*) soybean line JD321, its recipient soybean variety Jack and JD321 treated with glyphosate (JD321G), were all used to assess the alterations in the root‐associated microorganism and nutrient dynamics in response to both genetic modification and glyphosate application. Soil samples were collected at the flowering, seed filling and maturing stages, as well as three rhizosphere host niches from the interior to the outside of the roots, including intact roots (RT), rhizospheric soil (RS) and surrounding soil (SS). We aimed to test the following hypotheses: (i) the GM soybean with insect‐resistance and glyphosate‐tolerance, as well as glyphosate application would alter the rhizosphere microbial communities and nutrient dynamics; (ii) host niches would determine the differentiation of root‐associated microbial communities in response to newly introduced genes and glyphosate application.

## EXPERIMENTAL PROCEDURES

### Plant materials, sampling methods and DNA extraction

In this study, the control recipient soybean cultivar Jack and GM soybean JD321 (produced by the insertion of the *g2m‐epsps*, *gr79m‐epsps* and *cry1c* genes) were selected as the plant materials. The experimental field was at Shunyi Experimental Field Station of the Crop Research Institute (Institute of Crop Sciences, the Chinese Academy of Agriculture Sciences, CAAS), Beijing, China (N 40.229°, E 116.554°). According to the information obtained from the China Soil Database (http://vdb3.soil.csdb.cn/), the soil type belonged to the aquic cinnamon soil subgroup. JD321 with glyphosate treatment was named as JD321G, and the plots were sealed by spraying glyphosate (900 g hectare^−1^) on 24 June 2019. The experimental samples were collected before planting (4 June 2019), at the flowering stage (16 July 2019), at the seed filling stage (21 August 2019) and at the maturing stage (16 October 2019) respectively.

Each soybean cultivar was collected from three plots (10 × 15 m per plot) as three replicates randomly distributed over the field. In each plot, five plants were collected in the centre according to the diagonal intersection as samples. Thus, the total number of plants collected is 45 (15 control, 15 transgenic and 15 transgenic with glyphosate treatment) in one period. To avoid false environments, the roots and soil that were at the interface were avoided (Peiffer et al., 2013). After excavation, plants and soil were placed in a plastic bag with several pre‐freezing chemical ice packs. Bulk soils (BS) were sampled before planting. Surrounding soils (SS) were soil loosely adhering to the roots, and were collected by shaking off the soybean plant. Rhizospheric soils (RS) were then collected after brushing with phosphate‐buffered saline (PBS). The roots (RT) were then further washed with PBS and centrifuged at 4000 *g*, and the samples were collected by grinding with liquid nitrogen and defined as RT samples (a mixed sample of the rhizoplane and endosphere; Yang et al., 2020). These sampling steps were done using the method described by previous studies with modifications (Inceoglu et al., 2010; Lu et al., 2018; Wen et al., 2019). After taking all these samples to the laboratory, the plants and soil samples were then stored at 4°C for a basic physicochemical analysis and at −80°C for DNA extraction.

The bulk soils used to determine the total soil organic carbon (C) and total nitrogen (N) content, and rhizospheric soils used to detect the enzyme activities, were all filtered through a 50‐μm mesh sieve after natural air drying. The enzyme activities of nitrate reductase (S‐NR, Art. No. BC3105, EC 1.7.99.4), nitrite reductase (S‐NiR, Art. No. BC2995, EC 1.7.99.3), urease (S‐UE, Art. No. BC0125, EC 3.5.1.5), sucrase (S‐SC, Art. No. BC0245, EC 3.2.1.26), acid phosphatase (S‐ACP, Art. No. BC0285, EC 3.1.3.2) and alkaline phosphatase (S‐AKP/ALP, Art. No. BC0145, EC 3.1.3.1) were measured by corresponding kits purchased from Solarbio, China. The total C and N content of plants and soil were measured in the Center of Modern Analysis, Nanjing University, by using air‐dried soils and the Perkin‐Elmer 240c analyser.

DNA for metagenomic analysis was extracted from approximately 0.3 g of all these samples by using the PowerSoil DNA Isolation Kit (MoBio Laboratories Inc., Carlsbad, CA, USA). The experimental method also follows the instructions of previous studies (Lu et al., 2017; Wen et al., 2019, 2020). Before we quantified the concentration of each metagenomic DNA by using a Qubit Fluorometer (Qubit 2.0, Invitrogen, Carlsbad, USA) and ensured it was more than 10 ng/μl, the DNA samples were first assessed for quality on a 1% agarose gel (Kennedy et al., 2014).

### DNA amplicon sequencing via Illumina MiSeq platform

We used high‐throughput sequencing and amplicons of approximately 468 bp encompassing the V3‐V4 hypervariable regions of the 16 S rRNA and approximately 350 bp encompassing the ITS1 region of the internal transcribed spacer. The primers of V3–V4 region are forward primer 338F (5′‐ACTCCTACGGGAGGCAGCAG‐3′) and reverse primer 806R (5′‐GGACTACHVGGGTWTCTAAT‐3′; Xu et al., 2016), while the primers of ITS1 region are forward primer ITS1F (5′‐CTTGGTCATTTAGAGGAAGTAA‐3′) and reverse primer ITS2R (5′‐GCTGCGTTCTTCATCGATGC‐3′; Adams et al., 2013). The samples were sent to Majorbio Bio‐pharm Technology Co., Ltd (Shanghai, China) to perform PCR amplification, product purification, library quality determination and high‐throughput sequencing of the qualified libraries on the Illumina MiSeq platform (Illumina, CA, USA) with the MiSeq Reagent Kit. A total of 81 16 S rRNA sequencing clean data and 81 ITS sequencing clean data have been submitted to the SRA, and the SRA accession numbers are PRJNA664889 and PRJNA665106 respectively.

### Analyses of amplicon sequencing data

The analysis of 16 S rRNA amplicon sequencing data based on the database silva138/16s (http://www.arb‐silva.de) with sub‐sampled used the software R (v3.1.3) in platform I‐Sanger (http://www.i‐sanger.com) according to the minimum sample number of sequence, and eliminated the data of phylum Cyanobacteria and family Mitochondria to avoid the interference of mitochondrial and chloroplast genes. Additionally, the analysis of ITS amplicon sequencing data is based on the databases Unite8.0/ITS_fungi (http://unite.ut.ee/index.php) and also sub‐sampled according to the minimum sample number of sequences.

Alpha Diversity was analysed through observed species, chao1, ACE, shannon, simpson and coverage indices while Beta Diversity was analysed through principal component analysis (PCA), principal co‐ordinates analysis (PCoA), non‐metric multidimensional scaling analysis (NMDS) and partial least squares discriminant analysis (PLS‐DA; Caporaso et al., 2010; Schloss et al., 2009). The software PICRUSt and Fungi Functional Guild (FUNGuild) were used to analyse the functional genes of the bacterial community and fungal community respectively. LefSe analysis was performed according to all‐against‐all, and only taxa with a significant LDA threshold value of >4 were represented. All these analyses were performed on the platform I‐Sanger (https://cloud.majorbio.com; Wen et al., 2020).

### Quantification of *nifH* by quantitative real‐time PCR

To compare the relative abundance of the *nifH* gene, a quantitative real‐time PCR (qPCR) assay was performed. The primer pair of PolF–PolR was used for the quantification of the *nifH* gene and the primer pair of 338F − 518R for the 16 S rRNA was used as an internal control (Poly et al., 2001; Yang et al., 2012). The final 20 μl reaction volume contains 50 ng DNA (∼1 μl), 1 μl primer pair (5 pM) and 10 μl of 2× SYBR Green mixture (Roche, Switzerland). Each group contained three biological replicates (i.e., three DNA samples), and each DNA sample contained three technical duplicates. The PCR program contained 95°C for 10 min, followed by 40 cycles consisting of 95°C for 15 s and 60°C for 1 min (Wen et al., 2019).

### Statistical analyses

One‐way ANOVA was used to evaluate the selected properties of soils and plants, alpha diversity indices, the quantification of *nifH* gene, and the abundance of species and functional genes by using software GraphPad Prism 8. The analysis of similarities (ANOSIM) and PERMANOVA analysis (Adonis) were performed by using the software R (v3.1.3) in platform I‐Sanger based on the Bray–Curtis distance metric (Zhou et al., 2011).

## RESULTS

### Properties of soils and plants

The soil pH ranged from 7.1 to 7.5, and at the same sampling stage, no significant differences among the three treatments were found (Table 1). However, the growth of soybean at four different sampling stages dramatically elevated the pH values in JD321G. Compared to JD321 and JD321G, Jack had higher soil N contents before planting and during the flowering stage, but JD321 and JD321G had higher soil C:N ratios. The soil water and C content had no significant difference among Jack, JD321 and JD321G at the same sampling stage (Table 1). Additionally, for the three treatments, the C content of the soybean plant was lower during the flowering stage than during the seed filling and maturing stages, but the N and C content of the plants did not differ among Jack, JD321 and JD321G (Table 1).

**TABLE 1:** Physicochemical properties of soil and plant

	Sampling stages	Before planting (mean ± SD)			Flowering stage (mean ± SD)				Seed filling stage (mean ± SD)			Maturing stage (mean ± SD)		
	Samples	Jack	JD321	JD321G	Jack	JD321	JD321G		Jack	JD321	JD321G	Jack	JD321	JD321G
Soil	pH	7.28 ± 0.16	7.28 ± 0.04	7.14 ± 0.08^A^	7.24 ± 0.07	7.20 ± 0.16	7.26 ± 0.09 ^AB^		7.22 ± 0.19	7.45 ± 0.06	7.44 ± 0.03 ^B^	7.39 ± 0.04	7.51 ± 0.04	7.53 ± 0.14^B^
	Water content (%)	15.92 ± 0.51^A^	16.72 ± 0.33 ^A^	16.74 ± 0.24 ^A^	11.27 ± 1.59 ^B^	11.54 ± 0.82^B^	12.18 ± 0.70 ^B^		23.41 ± 0.80^C^	22.43 ± 0.63 ^C^	22.44 ± 0.30 ^C^	18.97 ± 0.55^D^	19.43 ± 0.20 ^D^	19.13 ± 0.28 ^D^
	C content (%)	0.95 ± 0.02	1.00 ± 0.02	1.00 ± 0.02	1.01 ± 0.02	0.94 ± 0.01	0.94 ± 0.04		1.00 ± 0.06	0.93 ± 0.05	0.97 ± 0.01	0.98 ± 0.10	0.91 ± 0.03	0.96 ± 0.03
	N content (%)	**0.11 ± 0.01** ^**a/** A^	**0.09 ± 0.01** ^**b**^	**0.09 ± 0.00** ^**b**^	**0.12 ± 0.01** ^**a/** A^	**0.09 ± 0.01** ^**b**^	**0.08 ± 0.01** ^**b**^		0.08 ± 0.01^B^	0.08 ± 0.01	0.08 ± 0.01	0.08 ± 0.01^B^	0.08 ± 0.01	0.09 ± 0.01
	C:N ratios	**8.39 ± 0.37** ^**a/** A^	**11.57 ± 0.83** ^**b**^	**11.11 ± 0.59** ^**b**^	**8.53 ± 0.34** ^**a/** A^	**10.13 ± 0.56** ^**b**^	**11.38 ± 1.19** ^**b**^		11.79 ± 1.46^B^	11.23 ± 0.81	11.63 ± 0.64	11.83 ± 1.76^B^	11.94 ± 0.56	11.07 ± 0.77
Plant	C content (%)	NA	NA	NA	39.41 ± 1.79 ^A^	39.52 ± 1.04 ^A^	37.92 ± 0.00 ^A^		42.39 ± 0.37^B^	43.03 ± 0.39 ^B^	43.14 ± 0.18 ^B^	42.78 ± 0.86^B^	43.18 ± 0.46 ^B^	43.24 ± 0.25 ^B^
	N content (%)	NA	NA	NA	3.47 ± 0.18	3.37 ± 0.36	3.19 ± 0.13		2.50 ± 0.28	2.57 ± 0.12	2.66 ± 0.54	2.63 ± 0.95	2.91 ± 0.56	3.23 ± 0.92

*Note*: SD represents standard deviation. Values were mean ± SD (*n* = 3). C content and N content represent carbon content and nitrogen content. Jack, JD321 and JD321G represent the control soybean line Jack, the GM soybean line JD321 and JD321 with glyphosate treatment, respectively. Soil analysis using the samples bulk soils before planting and surrounding soils at the flowering, seed filling and maturing stages. The significance test was performed by using one‐way ANOVA. The values in bold with different lowercase letter superscripts indicate the significant difference (*p* < 0.05) between Jack, JD321 and JD321G groups by the tests, while the values with different capital letter superscripts indicate the significant difference between different sampling stages groups by the tests.

**FIGURE 1:**
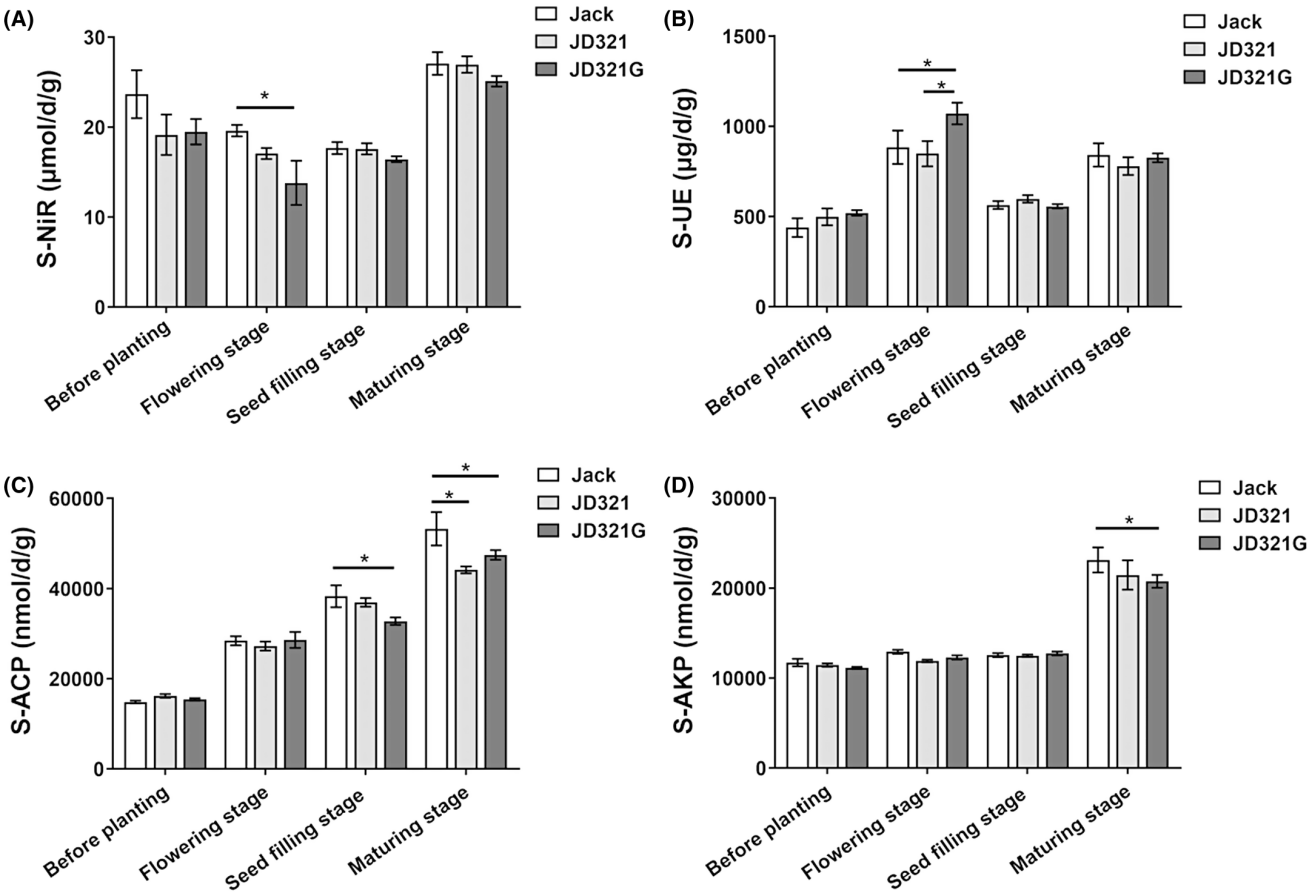
The activities of four key enzymes involved in the nitrogen and phosphorus cycle of root‐associated microbial communities. The activities of nitrite reductase (A), urease (B), acid phosphatase (C) and alkaline phosphatase (D) in rhizospheric soil were analysed by using one‐way ANOVA. Jack, JD321 and JD321G represent the control soybean line Jack, the GM soybean line JD321 and JD321 with glyphosate treatment, respectively.

Compared with Jack, lower nitrite reductase and higher urease activities were measured in the rhizospheric soils of JD321G at flowering stage, meanwhile lower acid and alkaline phosphatase activities were also observed at maturing stage (Figure 1). There was no significant difference in the enzyme activities of sucrase and nitrate reductase among the three treatments (Figure S1–S12). It is worth mentioning that the enzyme activities also significantly changed at different sampling stages. For example, the nitrate reductase and urease activities were higher at flowering stage, while the nitrite reductase, acid and alkaline phosphatase activities were higher at maturing stage (Figure 1 and Figure S1–S12).

### Alpha and Beta diversity of soybean root‐associated microbial communities

**FIGURE 2:**
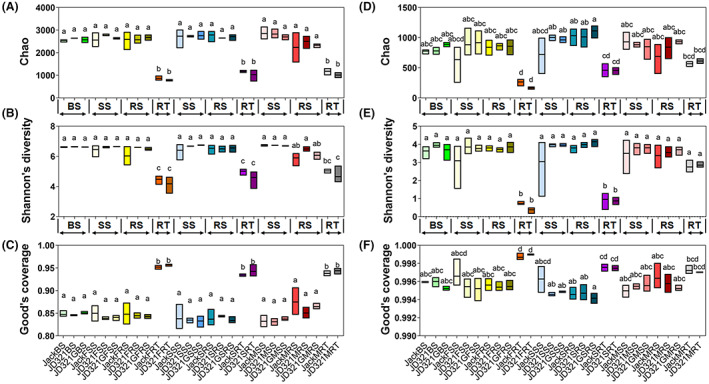
The boxplot of alpha diversity of root‐associated microbial communities. The results of alpha diversity were divided into two groups through three different indices (chao value, Shannon value and Good's coverage) according to different sequencing results. These are the alpha diversity of bacterial community (A, B and C) and fungal community (D, E and F). Jack, JD321 and JD321G represent the control soybean line Jack, the GM soybean line JD321 and JD321 with glyphosate treatment respectively. F, S and M represent the flowering, seed filling and maturing stages respectively. BS means bulk soils which sampled before planting. SS, RS and RT represent surrounding soils, rhizospheric soils and intact roots respectively. The statistical analysis of these indices was calculated by using one‐way ANOVA.

The sequencing data were sufficient and reliable (Figures S2 and S3), with 18/15 core species, and 7497/3689 common species in 16 S rRNA/ITS sequencing samples (Table S1–S3). The total shared number of OTUs of rhizospheric soils gradually decreased with the growth of soybean (Figures S4 and S5). No significant difference in alpha diversity (i.e. all pair‐comparison showed *p* > 0.05) was found among Jack, JD321 and JD321G in bacterial and fungal communities (Figure 2 and Figure S6). Moreover, soil niches (i.e. SS and RS) had higher community richness and diversity, and had lower community coverage than niche RT (Figure 2 and Figure S6).

**FIGURE 3:**
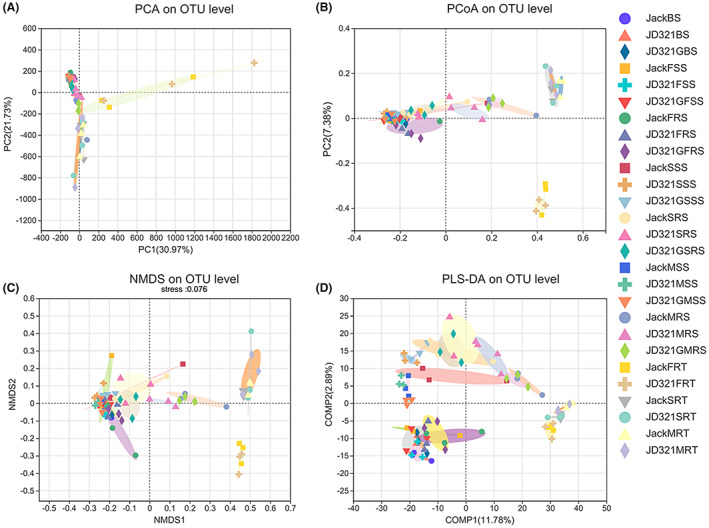
PCA, PCoA, PLS‐DA and NMDS on OTU level of bacterial community. (A) PCA based on OTU abundance of bacterial community. (B) PCoA based on Bray–Curtis distance of bacterial community. (C) NMDS based on Bray–Curtis distance of bacterial community. (D) PLS‐DA based of bacterial community. See the treatment details in the figure legend of Figure 2.

The PCA, PCoA, NMDS and PLS‐DA on OTU level showed no significant distinction in distance among Jack, JD321 and JD321G at same host niches of soybean rhizosphere in bacterial and fungal communities (Figure 3 and Figure S7). Notably, there existed significant distinction in distance between niche RT and other soil niches (Figure 3 and Figure S7). Comparison based on family level got the same results as that of OTU level (Figure S8). The statistical analyses of ANOSIM and Adonis proved that there existed no significant difference among Jack, JD321 and JD321G groups in the same host niches and at same sampling stages (Table S2). Moreover, the overall statistical results by using the combined groups showed that there existed significant differences among different host niches of soybean rhizosphere and different sampling stage, while there was no significant difference among Jack, JD321 and JD321G groups (Table 2).

**TABLE 2:** Significance tests of overall microbial communities between samples

Group vs. Group	Bacterial community				Fungal community			
	Adonis		ANOSIM		Adonis		ANOSIM	
	*R* ^2^	*p‐*value	Statistic	*p‐*value	*R* ^2^	*p‐*value	Statistic	*p‐*value
Jack vs. JD321	0.0254	0.311	−0.0039	0.461	0.0286	0.217	−0.0019	0.449
Jack vs. JD321G	0.0185	0.775	−0.0247	0.945	0.0305	0.189	0.0071	0.244
JD321 vs. JD321G	0.0154	0.996	−0.04	0.996	0.0168	0.743	−0.0178	0.828
Flowing vs. Seed filling	0.0595	**0.017**	0.1224	**0.006**	0.0263	0.242	0.0385	0.099
Flowing vs. Maturing	0.0765	**0.008**	0.1564	**0.004**	0.1097	**0.002**	0.1712	**0.002**
Seed filling vs. Maturing	0.0391	0.086	0.0645	**0.049**	0.0701	**0.016**	0.1003	**0.02**
BS vs. SS	0.0475	**0.005**	−0.0765	0.712	0.0357	0.164	−0.0411	0.591
BS vs. RS	0.1061	**0.001**	0.02	0.323	0.1110	**0.005**	0.0206	0.34
BS vs. RT	0.4603	**0.001**	0.9899	**0.001**	0.6555	**0.001**	1	**0.001**
SS vs. RS	0.1066	**0.001**	0.2983	**0.001**	0.0923	**0.001**	0.1519	**0.001**
SS vs. RT	0.4712	**0.001**	0.9909	**0.001**	0.5857	**0.001**	0.9288	**0.001**
RS vs. RT	0.2941	**0.001**	0.7693	**0.001**	0.4790	**0.001**	0.909	**0.001**

*Note*: ANOSIM means analysis of similarities and Adonis is used for PERMANOVA (permutational multivariate analysis of variance) based on the Bray–Curtis distance metrics. The *p*‐values in bold indicate the significant difference (*p* < 0.05 [*]) between groups by the tests. BS means bulk soils sampled before planting. SS, RS and RT represent surrounding soils, rhizospheric soils and intact roots respectively. Other treatment's details are as in Table 1.

### Comparison of the compositions and functions of microbial communities

The composition of the major microbial taxa was then compared at different taxonomic levels. The results at phylum level showed that the relative abundance of Proteobacteria in the niche RT was higher than in soil niches, but the abundances of Acidobacteriota and Basidiomycota were significantly lower in RT compared to soil niches (Figure S9). There existed no significant difference among different treatments or among different sampling stages (Figure S9). Furthermore, using ternary plots to compare the composition of the microbiome at the genus level, it was discovered that there was a considerable variation across different host niches (Figure S10). The relative abundances of *Mortierella*, *Chaetomium* and *Pseudombrophila*, which belong to the families Mortierellaceae, Chaetomiaceae and Pyronemataceae, gradually increased from the inside to the outside of the root, while the abundances of genus *Chryseobacterium*, which belongs to the family Weeksellaceae, and *Streptomyces*, which belongs to the family Streptomycetaceae, gradually decreased (Figure S10). When the 10 identified species belonging to these five genera were examined further, only the abundances of *Mortierella elongate*, *Chaetomium piluliferum*, *Pseudombrophila hepatica*, *Chryseobacterium lathyri* and *Streptomyces scabiei* were consistent with the genus level trend, while other five species showed no difference among different host niches (Table S3).

**FIGURE 4:**
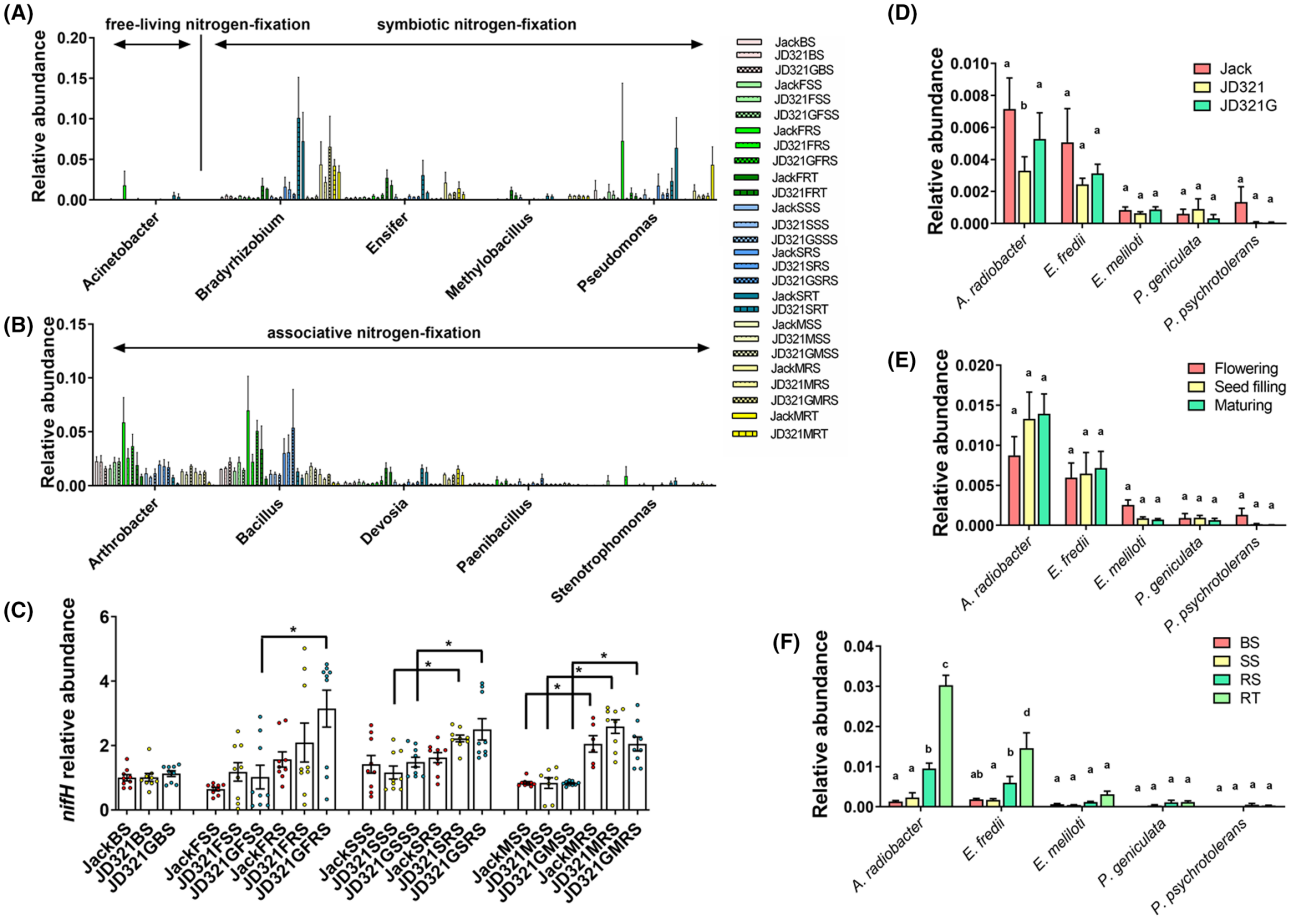
The composition and abundance of bacteria related to nitrogen fixation in soybean root‐associated bacterial community. (A) The abundance of free‐living and symbiotic nitrogen‐fixation bacteria at genus level. (B) The abundance of associative nitrogen‐fixation bacteria at genus level. (C) Relative abundance of *nifH* gene in soybean root‐associated bacterial community of Jack, JD321 and JD321G at different sampling compartments and stages, and the statistical analysis of these indices was calculated by using one‐way ANOVA. The abundance of nitrogen‐fixation bacteria at species level based on different treatment (D), different sampling stages (E) and different host niches (F), and the statistical analysis of these indices was calculated by using two‐way ANOVA. See the treatment details in the figure legend of Figure 2.

Further investigation of the compositions of bacterial genera associated to free‐living, associative and symbiotic nitrogen fixation revealed no significant difference in relative abundance of these nitrogen fixation bacteria among different treatments or sampling stages (Figure 4A,B). Notably, when compared to soil niches, the relative abundances of *Bradyrhizobium* and *Ensifer* were found to be significant higher in the niche RT (Figure 4A). The relative abundance of the *nifH* gene then revealed that there was no discernible difference across various treatments or stages, but that the *nifH* gene abundance in the niche RS was higher than that in the niche SS, especially at the maturing stage (Figure 4C). Lastly, five nitrogen‐fixation bacterial species were identified at the species level including *Agrobacterium radiobacter*, *Ensifer fredii*, *Ensifer meliloti*, *Pseudomonas geniculata* and *Pseudomonas psychrotolerans*. When compared to soil niches, *A. radiobacter*, *E. fredii* and *E. meliloti* were enriched in niche RT, but they all displayed no discernible difference in relative abundance among different treatments or stages (Figure 4D–F).

**FIGURE 5:**
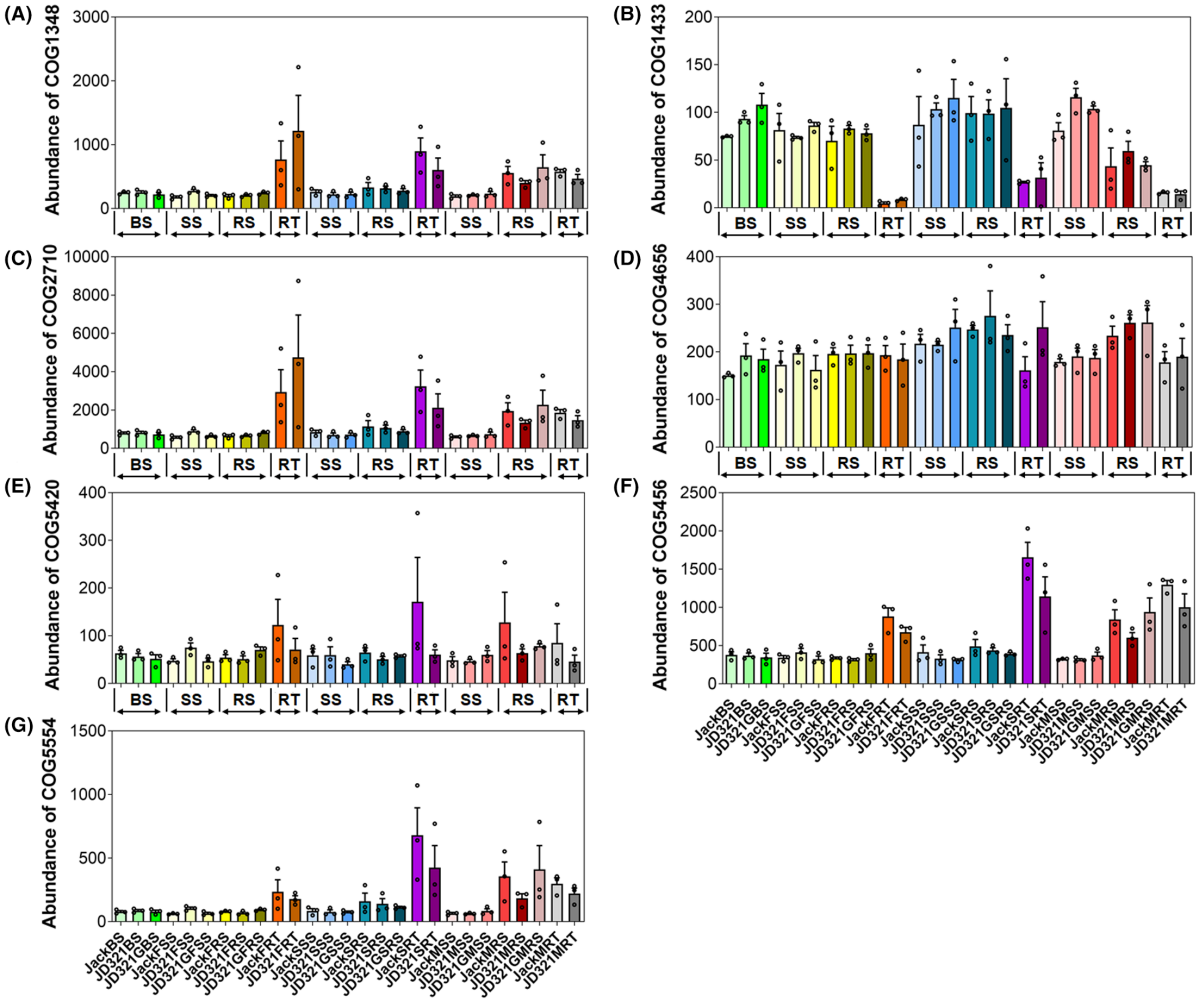
The relative abundances of COG function classification were directly related to nitrogen fixation. All these seven COGs from (A) to (G) were regarded as the key enzymatic reactions in nitrogen fixation, dinitrogenase iron‐molybdenum cofactor biosynthesis protein, nitrogenase, required for nitrogen fixation, nitrogen fixation, nitrogen fixation protein FixH and nitrogen fixation protein, respectively. See the treatment details in the figure legend of Figure 2.

A total of seven cluster of orthologous groups (COGs) function classification genes were found to be directly related to nitrogen fixation. Six of them, COG1348, COG2710, COG4656, COG5420, COG5456 and COG5554 were found to be enriched in RT when compared to soil niches (Figure 5). Only COG1433, which was identified as a dinitrogenase iron‐molybdenum cofactor biosynthesis protein, was found to be de‐enriched in RT (Figure 5). The cooccurrence network analysis plot at the OTU level revealed that the network complexity of the bacterial community decreased from the inside to the outside of the roots, however no difference was observed in the fungal community (Figure S11A,B). At phylum level, Patescibacteria was closely related to the niche RT at the seed filling and maturing stages, while Glomeromycota was closely related to JD321G in the niche RS during flowering and seed filling (Figure S11D–H). As shown in LefSe analysis, *Lysobacter soli* and *Penicillium kongii* in Jack, *Steriodobacter* and *Trichocladium asperum* in JD321 and *Chryseolinea* and *Pseudogymnoascus* in JD321G were their representative biomarker taxa, while different host niches also had their own representative biomarker taxa (Figure S12).

## DISCUSSION

### Host niches determine the differentiation of root‐associated microbial communities

Our results showed that the differentiation of root‐associated microbial communities was predominantly determined by the host niche, rather than newly imported genes or glyphosate application. These findings supported our second hypothesis that the sampling compartment (i.e. host niches) has a major impact on the assembly and the shift of bacterial and fungal communities, which was consistent with earlier findings (Anzalone et al., 2021; Loganathachetti et al., 2017; Wen et al., 2019; Yang et al., 2020). Different physical structures, biotic components and abiotic conditions create different potential niches for microbes (Aas et al., 2019). In this study, the abundance of PGPR and plant growth‐promoting fungi (PGPF) was changed gradually in the host rhizosphere niches from the inside to the outside of the soybean roots. Rhizobacteria related to nitrogen fixation represents the most important group of PGPR in soybean. *Bradyrhizobium* and *Ensifer*, two major nitrogen‐fixing genera, were more abundant in the host niche RT than RS (Figure 4A). The result implied that the soybean root had an enrichment effect on the nitrogen‐fixing genera, which was consistent with our previous findings (Wen et al., 2020). However, only two species, *E. fredii* and *E. meliloti* (Bellato et al., 1997; Galardini et al., 2013), were found to be enriched in RT, whereas no changes in abundance of *Bradyrhizobium* species were detected. Although the relative abundance of nitrogen‐fixating bacteria *Agrobacterium radiobacter* (Yablunenko et al., 1995) was higher in RT, the ternary diagram did not show the same trend of the genus *Agrobacterium* among different host niches. Such discrepancies might be due to insufficient sequencing depth or improper annotation (Zhang, Bai, et al., 2020). Furthermore, the absence of the genus *Agrobacterium* may be due to the reason that *Agrobacterium* in the 16 S SILVA taxonomy, *Agrobacterium* is combined into a single genus ‘*Allorhizobium*‐*Neorhizbium*‐*Pararhizobium*‐*Rhizobium*’ with genera *Burkholderia* and *Rhizobium* (Lu & Salzberg, 2020), as shown in Figure S12A, thus, the limitation of existing database may affect the analysis of specific functional species. Lastly, the relative abundance of only one function classification gene, COG1433 increased from the inside to the outside of the soybean roots. This COG was defined as dinitrogenase iron‐molybdenum cofactor biosynthesis protein and was closely related to *nif* gene cluster (Stoeckel et al., 2008; Toepel et al., 2008). On the contrary, five of the seven COGs directly associated with nitrogen fixation were enriched in the niche RT (Figure 5). These results also suggest that host niches significantly affect the relative abundance of nitrogen‐fixing functional genes by enriching the aforementioned nitrogen‐fixing genera and species.

Plants can improve their health or enhance their resistance against biotic and abiotic stresses by affecting the assembly of rhizosphere microorganisms, especially by enriching plant growth‐promoting bacteria and fungi (Pieterse et al., 2014). Three PGPF genera (Hansen et al., 2013; Ozimek & Hanaka, 2020; Wang, Zhang, et al., 2017), *Mortierella*, *Chaetomium* and *Pseudombrophila* were found to be increased gradually from the inside to the outside of the roots (Figure S10), implying that soybean prefer to accumulate such fungi far from their roots. *Mortierella elongata* has the potential to symbiotically interact with PGPRs like *Burkholderia* in the rhizosphere soil (Uehling et al., 2019). Furthermore, some symbiotic PGPF, such as mycorrhizal fungi, have been shown to develop optimal dispersal networks in the legume rhizosphere soil to aid rhizobia enrichment (Pieterse et al., 2014; Zhang, Li, et al., 2020). These findings may help to explain the enrichment trends of the three PGPF genera in RS and SS as compared to RT. Additionally, it was discovered in the current investigation that the relative abundance of one PGPR genus *Chryseobacterium* increased from the outside to the inside of the roots (Figure S10), indicating that the root had an enrichment effect on this PGPF (Zhang et al., 2021). According to (Pieterse et al., 2014; Rudrappa et al., 2008), PGPR epiphytes have a tendency to form biofilms, which are multicellular communities encased inside an extracellular matrix. This may be what causes PGPR genera like *Chryseobacterium* to be more abundant in the niche RT. Therefore, the differentiation of root‐associated microbial communities was determined by the host niches, which may have been primarily influenced by the root barrier or root exudates (Salas‐Gonzalez et al., 2021; Sasse et al., 2018; Zhalnina et al., 2018).

### Effects of plant growth stage on the nutrient dynamics and microbial communities

One of the interesting findings of this study was that, in addition to host niches, soybean growth stages also played a more significant role in determining the assembly and shift of microbial communities than newly introduced gene and glyphosate application. The present investigation found significantly increased nitrate reductase and urease activities at the flowering stage (Figure 1 and Figure S1–S12), suggesting that the growth stages of soybean also affected the nutrient dynamics in soil. Previous research found seasonal patterns of nitrate reductase and nitrogen fixation in peanut (*Arachis hypogaea* L.), that is, leaf nitrate reductase activity declined rapidly while N fixation activity of root nodules increased post‐flowering (Sung & Sun, 1990), which was consistent with our findings. Nitrate is an important factor affecting symbiotic nitrogen fixation of leguminous crops. Increased nitrate content causes a decrease in root nodule respiration rate and nitrogenase activity (Vadez et al., 1996). Therefore, higher enzyme activity of nitrate reductase is beneficial to root nodule development and nitrogen fixation at the flowering stage. In our study, it was observed that the relative abundance of genus *Arthrobacter* was higher at flowering stage than any other sampling stages (Figure 4). According to certain reports, the *Arthrobacter* bacteria can assist eliminate biological nitrogen, such as nitrate nitrogen (He et al., 2017, 2020). Therefore, greater nitrate reductase activities may result from the enrichment effect of *Arthrobacter* in soybean rhizospheric soil at flowering stage, may lead to the higher nitrate reductase activities, which is beneficial to root nodule respiration and boosts the effectiveness of nitrogen fixation.

### GM soybean with glyphosate application altered the nutrient dynamics

Our results showed that GM soybean with insect‐resistance and glyphosate‐tolerance, as well as glyphosate application, only altered the rhizosphere nutrient dynamics, not the assembly and shift of microbial communities in the real scenario. In this study, the growth of Bt‐soybeans had a major impact on soil enzyme activities involved in nutrient cycling, which was in line with earlier research (Li et al., 2019; Mandal et al., 2020). First, we found that GM soybean JD321 with glyphosate treatment (JD321G) had lower nitrite reductase and higher urease activities than the control recipient soybean cultivar Jack at the flowering stage (Figure 1), suggesting that the GM soybean with glyphosate application decreased nitrogen loss and increased the inorganic nitrogen supply in soil. The nitrite reductase is one of the intracellular enzymes that participates in denitrification and can reduce the accumulation of nitrite nitrogen in the environment (Meakin et al., 2007). According to some previous studies, denitrification may sometimes accelerate under Bt crops by promoting nitrite reductase activities along with an increase in denitrifying bacteria (Li et al., 2019; Wu et al., 2004). However, other studies claimed that Bt crops had little to no impact on bacterial nitrite reductases (Szoboszlay et al., 2019). These results were inconsistent with our findings, and the possible explanation can be that we applied glyphosate in the planting of GM soybean in the real scenario, which was not completely consistent with the treatments in the previous studies. Urease enzymes can help with nitrogen mineralization by converting urea into inorganic nitrogen (Li et al., 2019). Higher urease activity may result in enhanced nitrogen cycling, which is consistent with recent research that demonstrated increased nitrogen mineralization under Bt crops (Li et al., 2019; Sarkar et al., 2009). The higher nitrogen cycling in soil cause the plants grow bigger and retain more N and take up more N, while their roots release more exudates to stimulate saprophytic microbes for mineralization at the same time. Soil enzymes are a sensitive indication of soil metabolic processes and fertility, and their activities are affected by soil chemical properties and microbial compositions under GM plants (Bennicelli et al., 2009; Bila et al., 2020; Li et al., 2019). However, utilizing high‐throughput sequencing analysis in this study, the nitrogen‐fixing endosymbiont genus *Bradyrhizobium*, which was closely related to rhizobial denitrification (Lopez et al., 2017), along with some copiotrophic ureolyric microbes such as Betaproteobacteria, Alphaproteobacteria, and Gammaproteobacteria which were associated with soil urease activity (Wang et al., 2019), showed no significant difference in abundance among Jack, JD321 and JD321G.

The glyphosate application accelerated soil nitrogen cycling. When comparing JD321G with JD321 in this study, we discovered that the use of glyphosate increased the urease activity at the flowering stage (Figure 1), which was consistent with previous studies (Kunanbayev et al., 2019; Sannino & Gianfreda, 2001). Glyphosate is moderately persistent in soil, and the decomposition rate should be closely related to the enzymatic activities of soil microorganisms (Gimsing et al., 2004; Veiga et al., 2001). But as of now, there are no changes between JD321 and JD321G in the relative abundance of the related microorganisms.

The rhizospheric soils of JD321G were discovered to have less alkaline phosphatase and acid phosphatase activity than Jack (Figure 1), which suggests that GM soybean cultivation with glyphosate application may limit phosphorus mineralization. Although several investigations discovered that enzyme activities remained unchanged in Bt crops (Mina & Chaudhary, 2012), numerous studies have shown that the response rates of phosphatase are often lower in Bt crops than in non‐Bt crops in field experiments, which may be due to Bt protein secretion (Li et al., 2019; Sarkar et al., 2009). Acid phosphatase and alkaline phosphatase are strongly associated with rhizosphere phosphorus (P) content and P cycle efficiency, hence promoting the mineralization of soil organic P (Wang et al., 2022). Additionally, the nutritive usage of glyphosate was associated with the absorption of phosphate, which means that glyphosate application increased the amount of nutritive phosphorus in soil (Drzyzga & Lipok, 2018; Ortiz et al., 2017). This excess supply of phosphorus brought on by glyphosate application may also be the cause of the inhibitory activity of phosphatase under JD321G. Furthermore, root‐associated microbial communities play a major role in controlling the amount of available soil P, in particular, arbuscular mycorrhizal fungi (AMF) improved the mineralization of soil organic P and have a positive impact on root phosphatase activities (Cordero & Datta, 2016; Ortiz et al., 2015; Wu et al., 2021). However, there were no significant differences in the relative abundance of AMF or other P‐solubilizing microorganism among Jack, JD321 and JD321G. As a result, we found that GM soybean showed a considerable response to glyphosate application in rhizosphere nutrient dynamics, despite having no influence on the root‐associated microbial communities.

### Realities and hopes of agricultural microbiome

Microbiota are essential components of the soil, driving biogeochemical cycles and promote plant growth and productivity, and contribute to the end of hunger (Hu et al., 2022; Zhu et al., 2021). Application of agricultural microbiome could make the food and agricultural system more efficient, resilient and sustainable than rely on synthetic chemical fertilizer technology (Hu et al., 2022). Over the past decade, due to the risk of biotic and abiotic stresses and agrochemicals, and the loss of efficacy of some agrochemicals and plant breeding programmes, the use of agricultural microbiome to sustainably increase agricultural production has received more and more attention from researchers to practitioners (Batista & Singh, 2021). By integrating recent advances in plant‐associated microbiome science methods including genomic, metabolic techniques, bioinformatics and synthetic community with classical microbiology, researchers can better explore the mechanism of soil microbiota (including many species that have yet to be cultured and characterized) (van der Voort et al., 2016; Vorholt et al., 2017). Furthermore, the long‐term experiments of the complex interactions among microorganisms (e.g. quorum sensing) in depth and connecting environmental and evolutionary microbiology will also be necessary for fully understand the potential the potential efficacy and mechanism of the application of agricultural microbiome (Corral‐Lugo et al., 2016; Espinosa‐Urgel, 2022). Thus, the agricultural microbiome will not only play an important role in ecological agricultural production but also contribute towards basic study, job creation and mitigation of the impacts of climate change (Batista & Singh, 2021).

There are several important limitations to the study as well. Both the soybean yield traits and the Bt protein residues secreted by GM soybean in soil were not studied in this investigation. The present study is expected to provide a first‐hand data and scientific basis of the selection and application of plant growth promoting bacteria/fungi in soybean rhizosphere in the future work. Due to the complex network of root‐soil‐microbe interactions, it is necessary to conduct long‐term studies to analyse the effects of GM soybean with insect‐resistance, glyphosate‐tolerance and glyphosate application on rhizosphere interactions and functional groups of microbes in order to obtain comprehensive data for evaluating the bio‐safety of GM crops on the soil environment in ecological agriculture.

## CONCLUSIONS

The results showed that the transgenic soybean line treated with glyphosate altered soil nutrient dynamics by increasing soil N nutrient availability and decreasing soil P nutrient availability, but had no significant effect on root‐associated microbial communities. Moreover, the host niches and soybean growth stages had greater influence on the assembly and shift of microbial communities than novel gene insertion and glyphosate application. Thus, the use of GM soybean and glyphosate application can be deemed as safe for the composition and function of root‐associated microbial communities in the present study. These studies will be useful for precise and safe control of GM crops and glyphosate in ecological agriculture.

## AUTHOR CONTRIBUTIONS

**Minkai Yang:** Data curation (lead); formal analysis (lead); methodology (equal); software (lead); writing – original draft (equal); writing – review and editing (equal). **Fuhe Luo:** Data curation (supporting); formal analysis (supporting); investigation (supporting); methodology (equal); software (supporting); supervision (equal); validation (equal); visualization (equal); writing – original draft (supporting). **Yuchen Song:** Data curation (supporting); formal analysis (supporting); investigation (supporting); methodology (supporting); software (supporting); validation (supporting); visualization (supporting); writing – original draft (supporting). **Shenglin Ma:** Formal analysis (supporting); supervision (supporting); validation (supporting); visualization (supporting). **Yudi Ma:** Formal analysis (supporting); supervision (supporting); validation (supporting); visualization (supporting). **Aliya Fazal:** Formal analysis (supporting); supervision (supporting); validation (supporting); visualization (supporting). **Tongming Yin:** Conceptualization (supporting); formal analysis (supporting); methodology (equal); project administration (supporting); supervision (supporting); visualization (supporting). **Guihua Lu:** Conceptualization (supporting); formal analysis (supporting); methodology (equal); project administration (supporting); supervision (supporting); visualization (supporting). **Shucun Sun:** Conceptualization (supporting); formal analysis (supporting); project administration (supporting); visualization (supporting). **Jinliang Qi:** Conceptualization (supporting); formal analysis (supporting); project administration (supporting); supervision (supporting); visualization (supporting). **Zhongling Wen:** Conceptualization (equal); formal analysis (supporting); funding acquisition (supporting); methodology (equal); project administration (equal); software (equal); visualization (equal); writing – original draft (equal); writing – review and editing (equal). **Yongchun Li:** Conceptualization (equal); formal analysis (equal); project administration (lead); software (equal); supervision (equal); validation (equal); visualization (equal); writing – original draft (equal). **Yonghua Yang:** Conceptualization (equal); funding acquisition (lead); project administration (lead); resources (lead); supervision (lead); writing – original draft (equal); writing – review and editing (equal).

## FUNDING INFORMATION

This work was supported by grants from the National Natural Science Foundation of China (31870495, 32101383), the China Postdoctoral Science Foundation (2022M711562), the National Important Science & Technology Specific Project (2016ZX08011‐003), and the Program for Changjiang Scholars and Innovative Research Team in University from the Ministry of Education of China (IRT_14R27).

## CONFLICT OF INTEREST

The Ministry of Agriculture of the People's Republic of China issued permissions for the locations. The field study did not involve endangered species. The authors have no financial conflicts of interest, no competing interests or other interests that might be perceived to influence the results and/or discussion reported in this paper to declare.

## Supporting information


Figures S1–S12

Tables S1–S3

## Data Availability

All clean sequencing data associated with this study are deposited in the SRA, and the accession numbers are PRJNA664889 and PRJNA665106.
