# Supplementary material for: The host niches of soybean rather than genetic modification or glyphosate application drive the assembly of root‐associated microbial communities
Source: Microb Biotechnol. 2022 Nov 6;15(12):2942–57. doi: 10.1111/1751-7915.14164 (PMC9733649; doi:10.1111/1751-7915.14164)
Supplement: Supplementary file 1 — Figures S1–S12 [file MBT2-15-2942-s001.docx]

**The host niches of soybean rather than genetic modification or glyphosate application drive the assembly of** **root-associated microbial communities**

**Supplementary Figures**


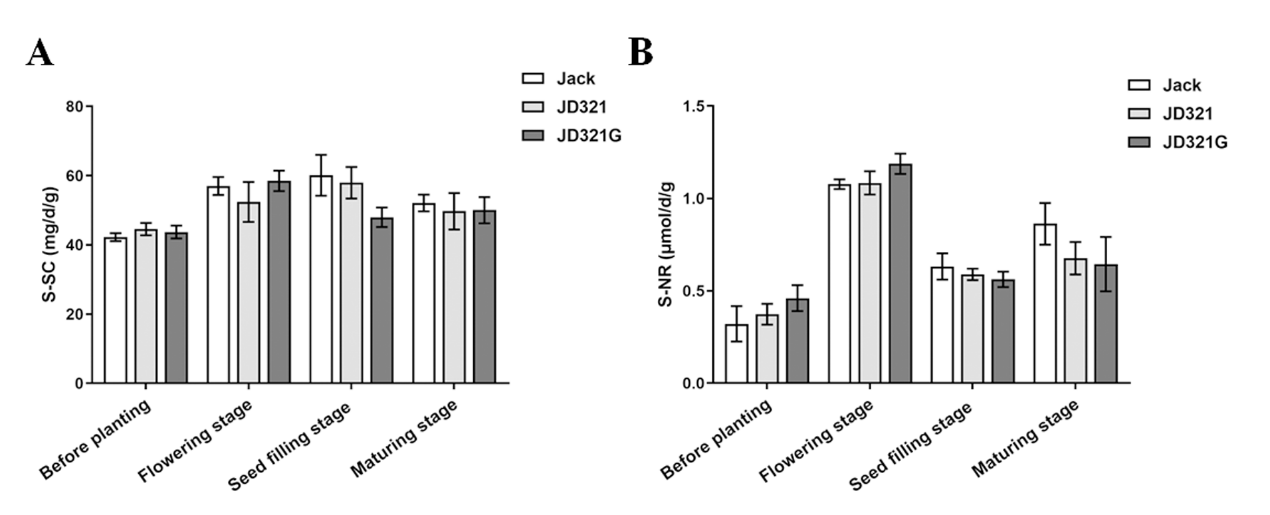


**Supplementary Figure 1** The activities of two key enzymes involved in the carbon and nitrogen cycles of root-associated microbial communities. Jack, JD321 and JD321G represent the control soybean line Jack, the GM soybean line JD321 and JD321 with glyphosate treatment, respectively.


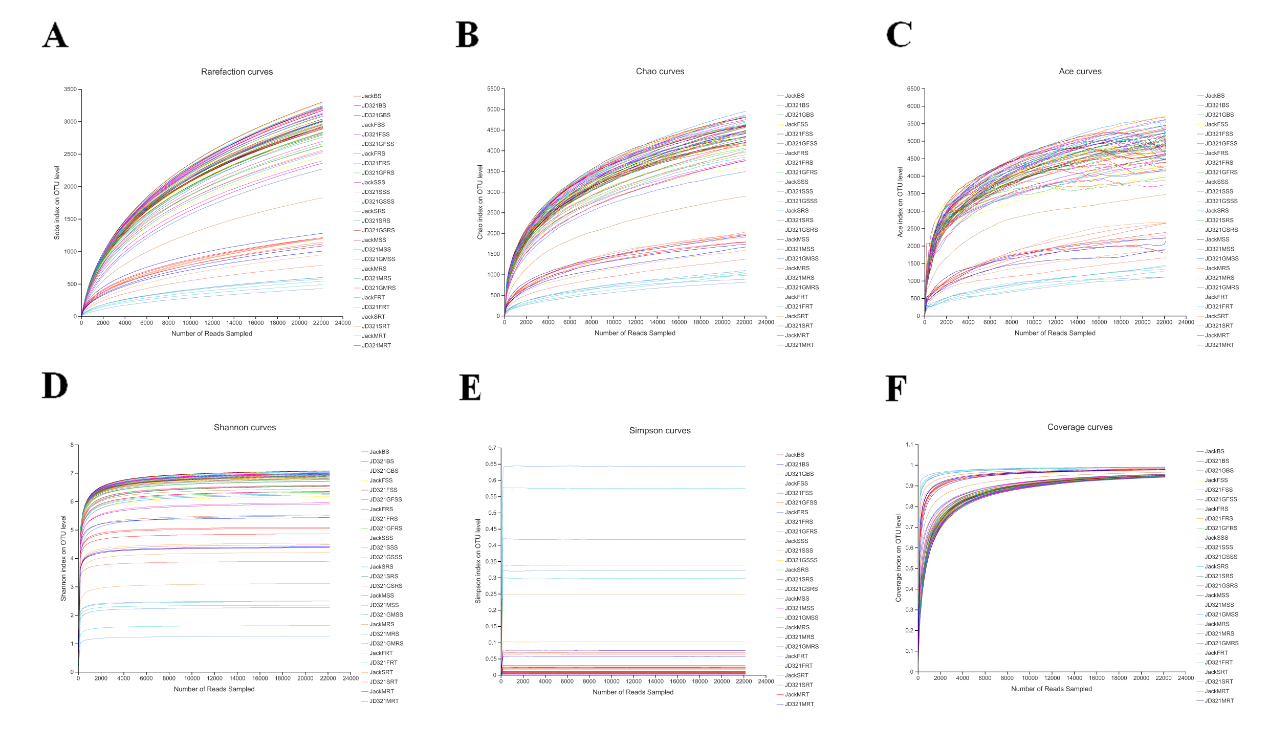


**Supplementary Figure 2** Rarefaction curve of 16S rRNA sequencing samples. Jack, JD321 and JD321G represent the untransformed control soybean line Jack, the genetically modified line JD321 and JD321 with glyphosate treatment, respectively. F, S and M represent the flowering, seed filling and mature stages, respectively. BS means bulk soils sampled before planting. SS, RS and RT represent surrounding soils, rhizospheric soils, and intact roots, respectively.


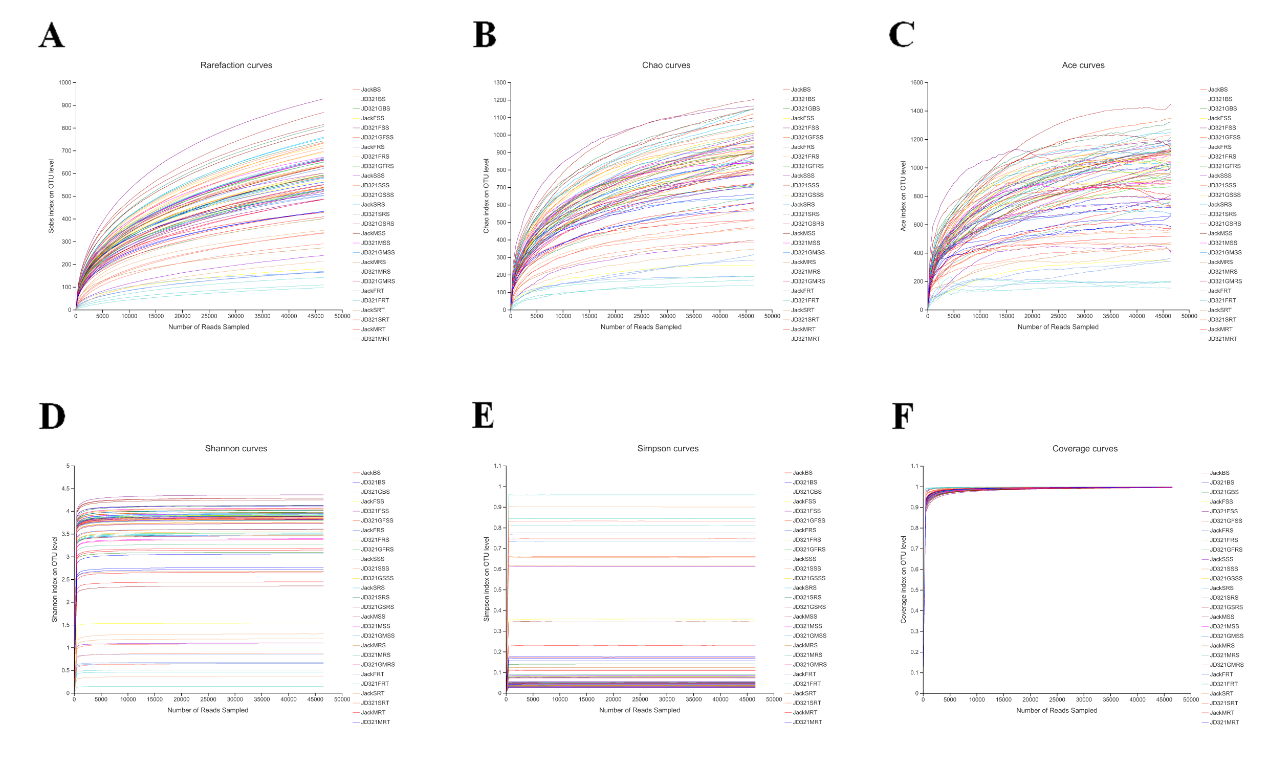


**Supplementary Figure 3** Rarefaction curve of ITS sequencing samples. See treatment details in Supplementary Figure 2.


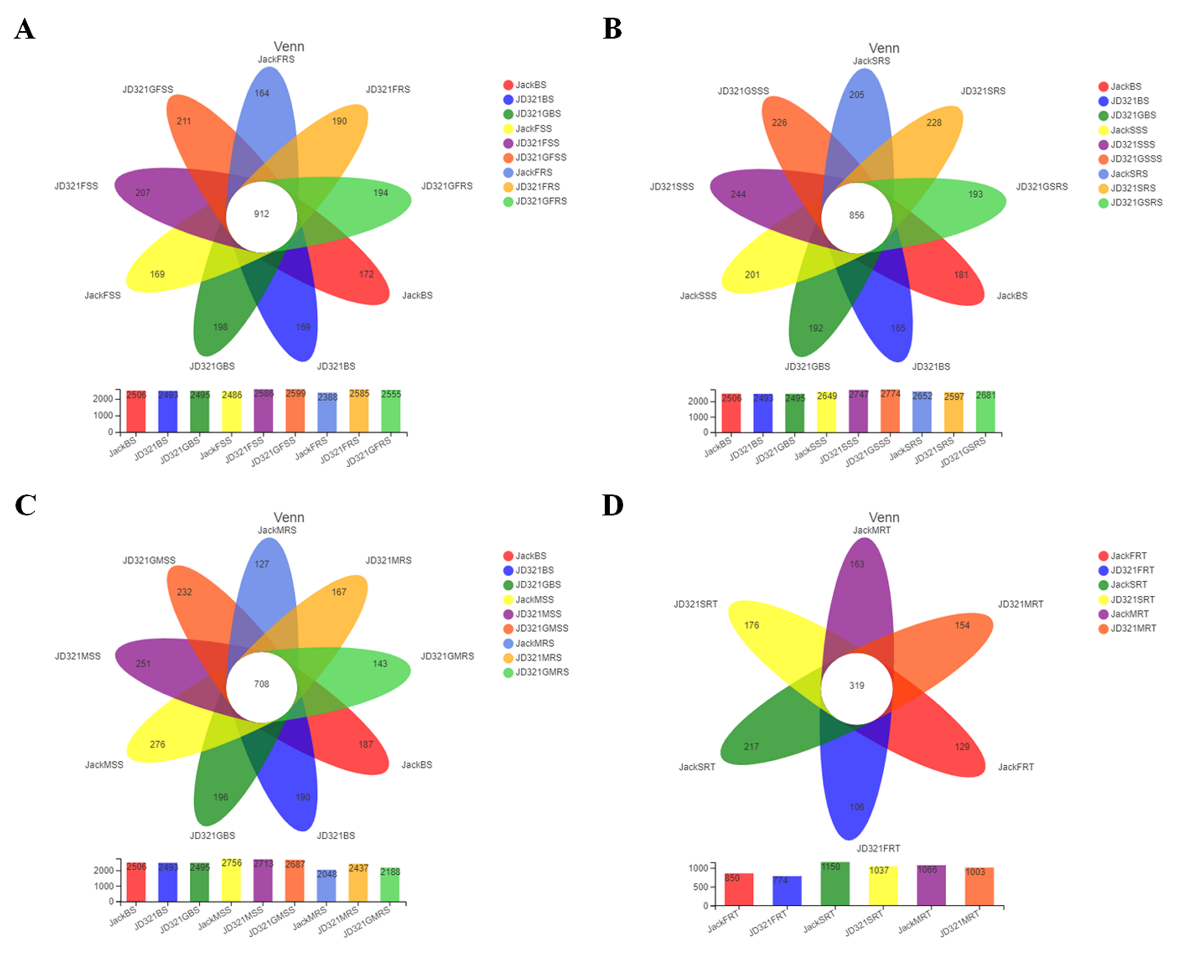
**Supplementary Figure 4** Venn diagram of 16S rRNA sequencing samples. The Venn diagram were plotted separately according to the sampling stages and sampling compartment. See treatment details in Supplementary Figure 2.


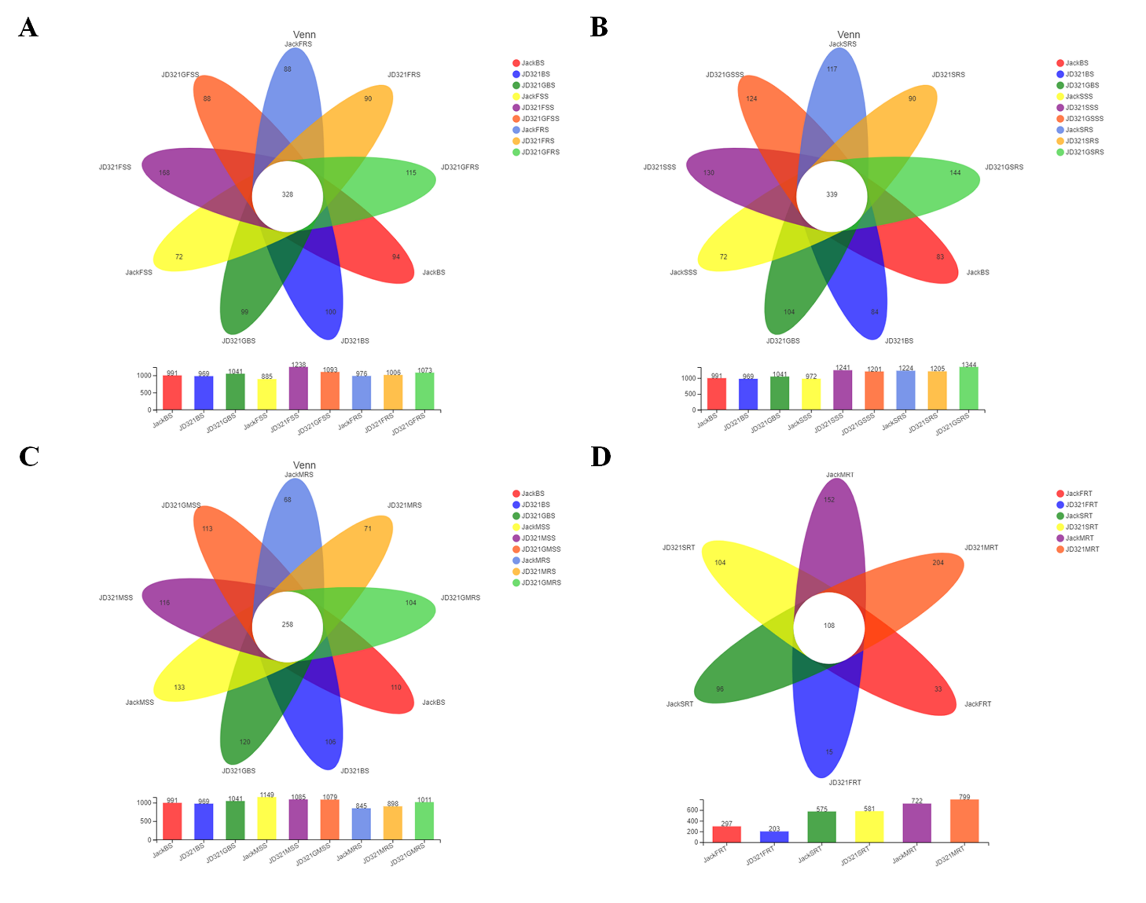


**Supplementary Figure 5** Venn diagram of ITS sequencing samples. The Venn diagram were plotted separately according to the sampling stages and sampling compartment. Treatment’s details were as in Supplementary Figure 2.


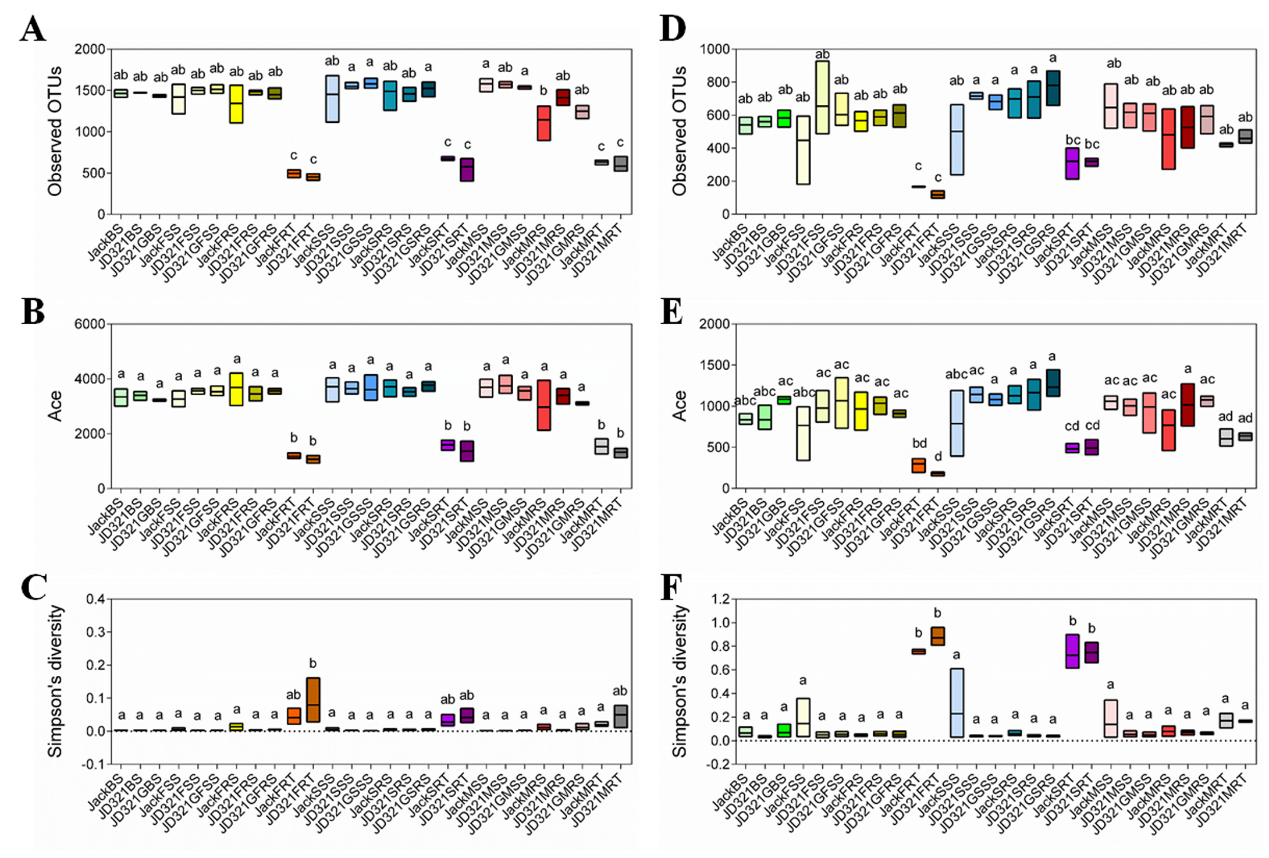


**Supplementary Figure 6** The boxplot of alpha diversity of root-associated microbial communities. The results of alpha diversity through three different indices (Observed OTU, Ace value and Simpson value) were divided in 2 groups according to different sequencing results. They are alpha diversity of bacterial community (A, B and C) and fungal community (D, E and F). See treatment details in Supplementary Figure 2.


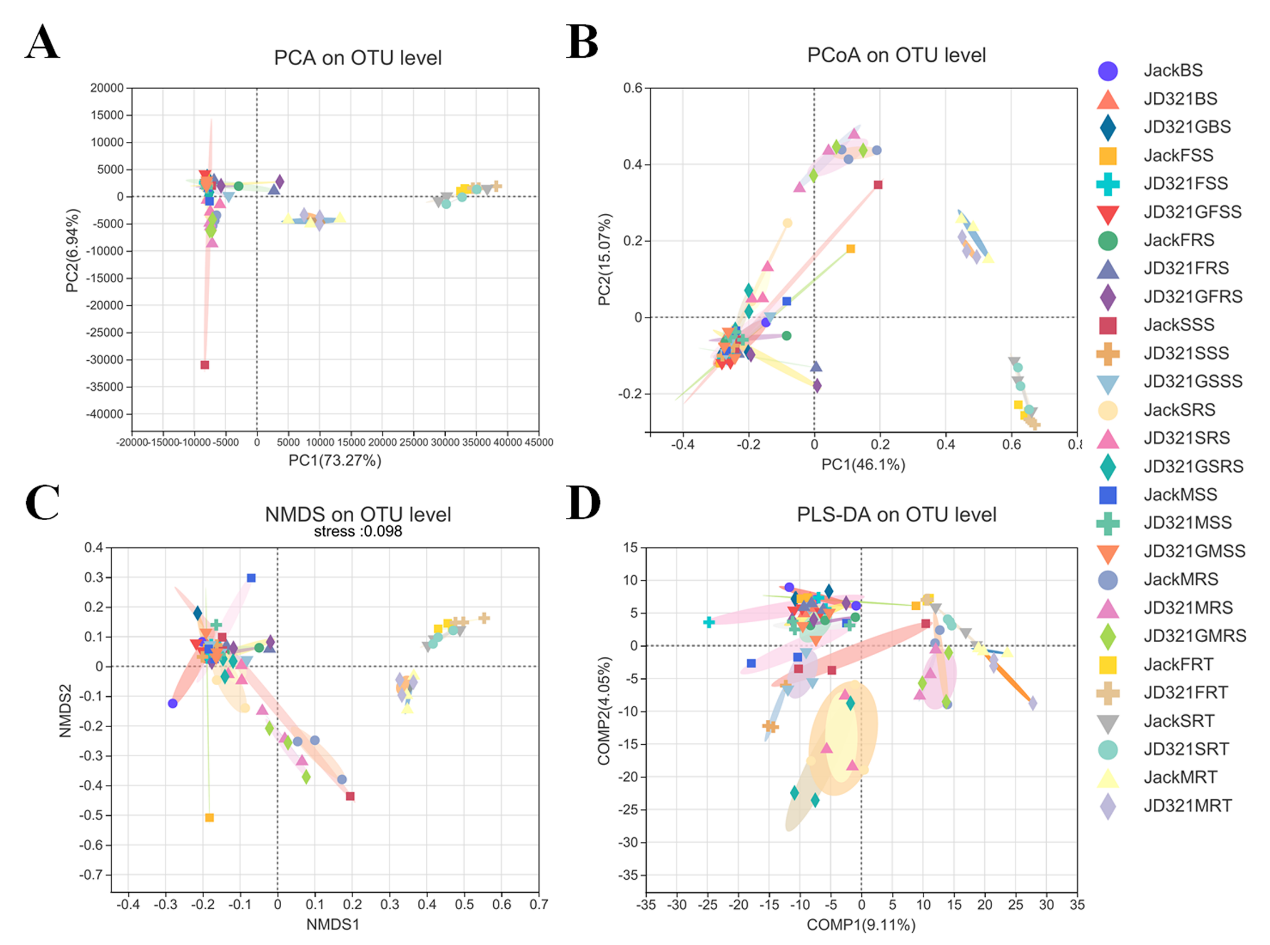


**Supplementary Figure 7** PCA, PCoA, PLS-DA and NMDS on OTU level of fungal community. (A) PCA based on OTU abundance of fungal community. (B) PCoA based on Bray-Curtis distance of fungal community. (C) NMDS based on Bray-Curtis distance of fungal community. (D) PLS-DA based of fungal community. See treatment details in Supplementary Figure 2.


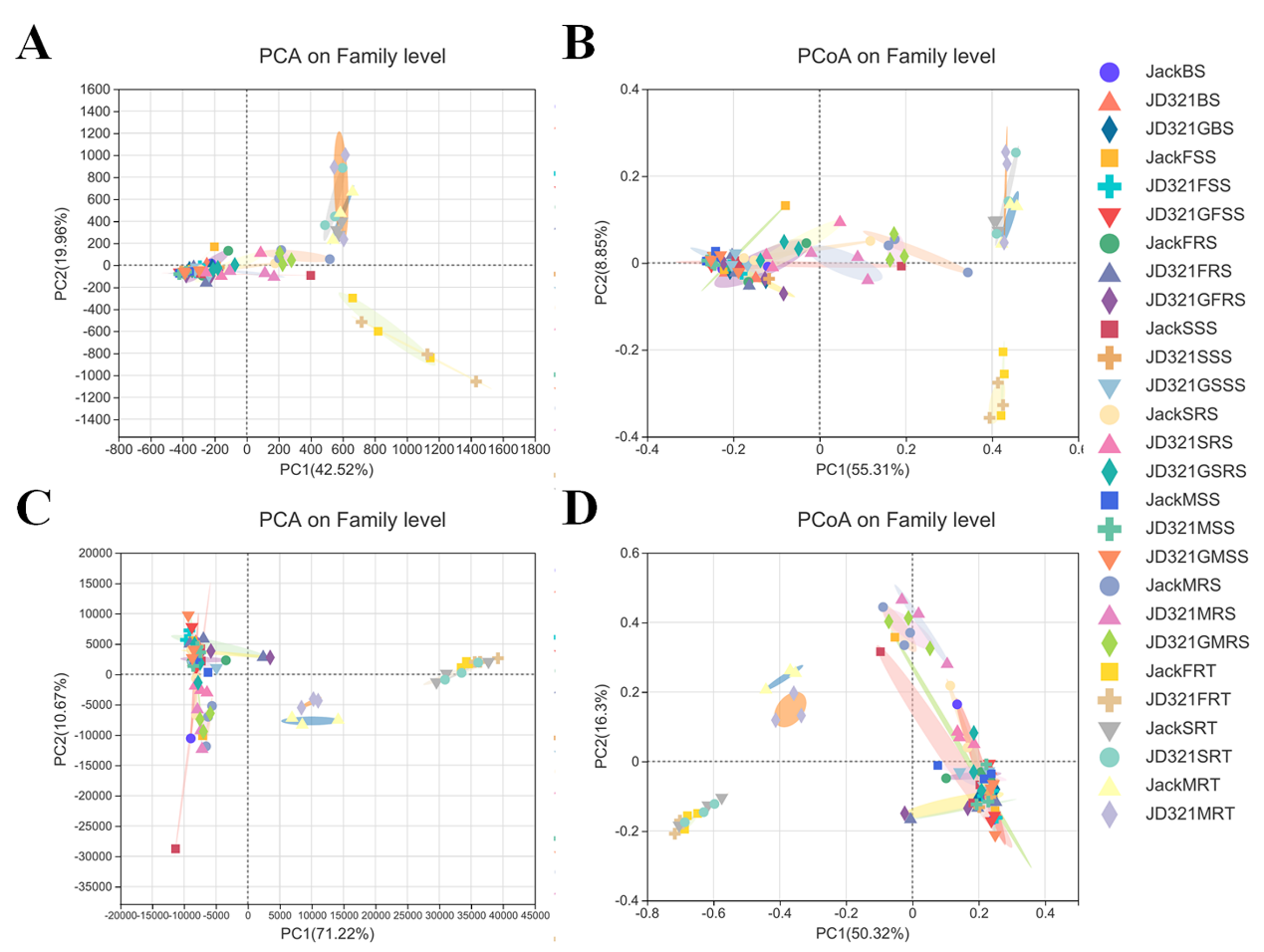


**Supplementary Figure 8** PCA and PCoA on family level. (A) and (B) were PCA and PCoA analyses of bacterial community. (C) and (D) were PCA and PCoA analyses of fungal community. See treatment details in Supplementary Figure 2.


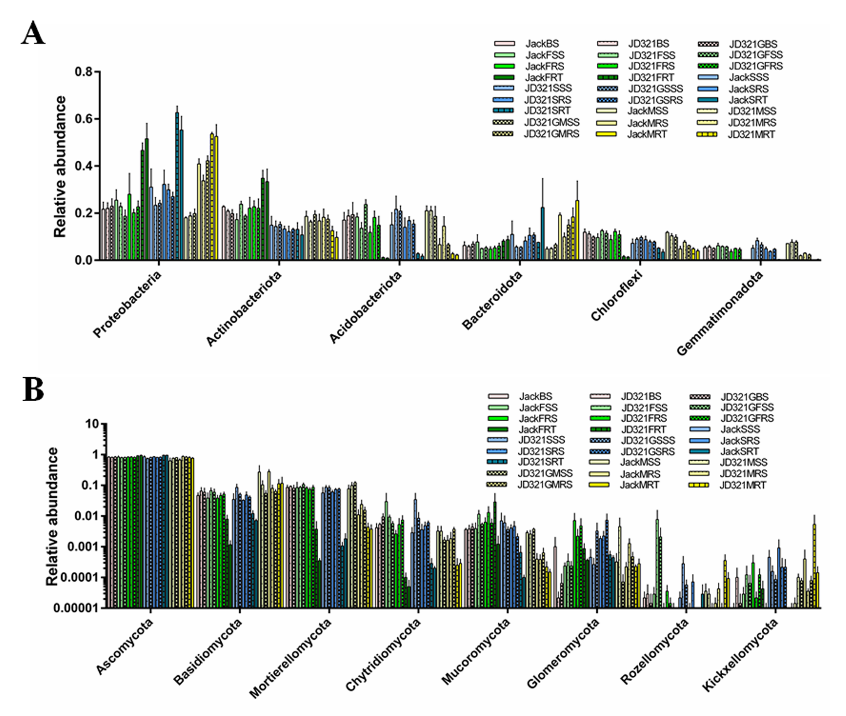


**Supplementary Figure 9** Relative abundances of major phyla of samples. (A) The relative abundances of six major phyla of bacterial community with abundances higher than 1%. (B) The relative abundances of eight major phyla of fungal community with abundances higher than 0.01%. See treatment details in Supplementary Figure 2.


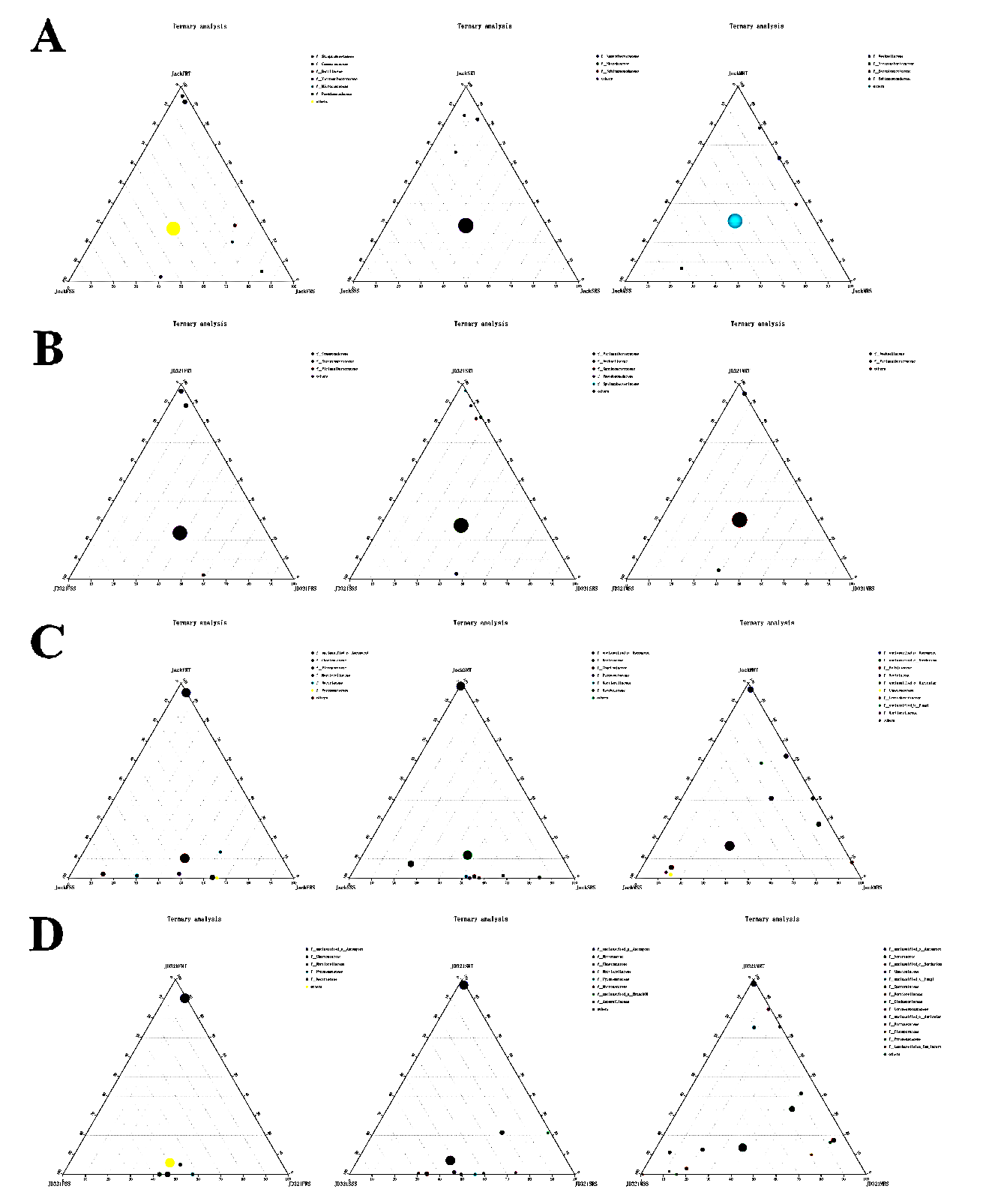


**Supplementary Figure 10** Ternary plot of the composition of root-associated microbial communities between groups roots, rhizospheric soils and surrounding soils. The composition of bacterial community (A and C) and fungal community (B and D) were making comparison at genus level with abundances higher than 5%. See treatment details in Supplementary Figure 2.


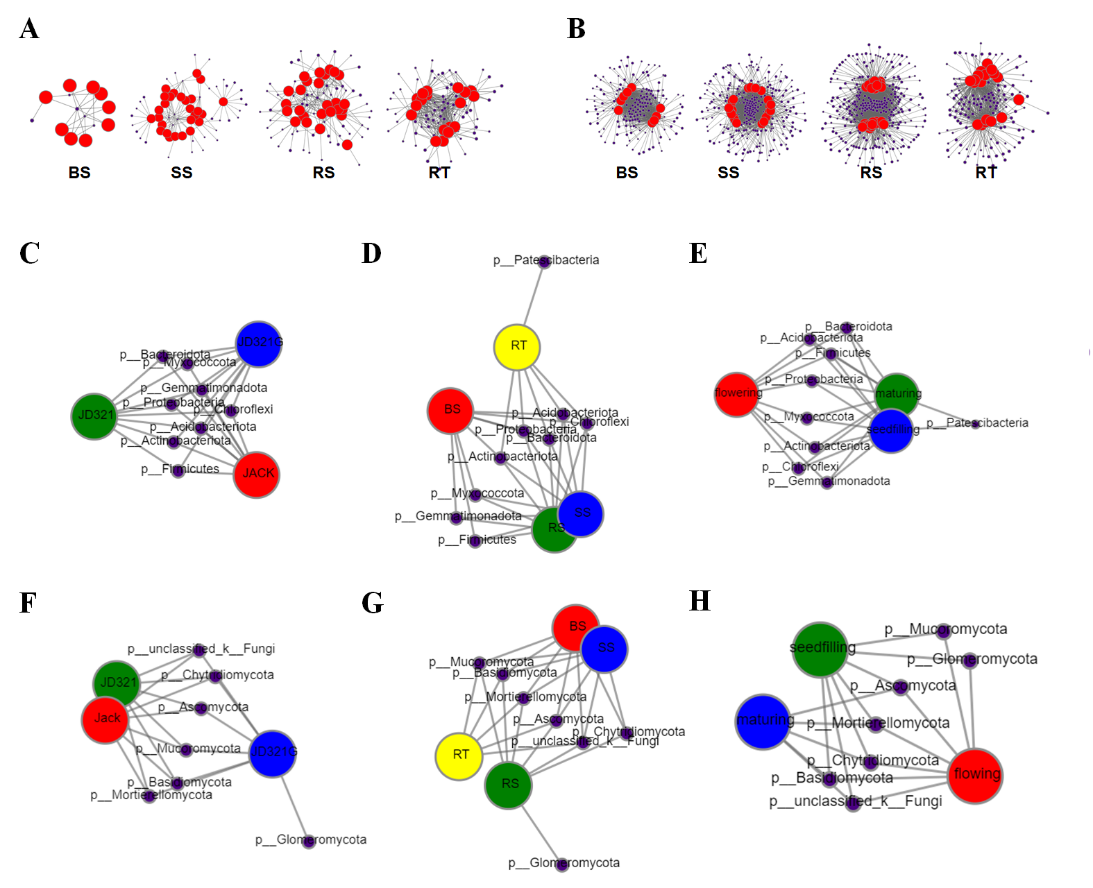


**Supplementary Figure 11** Network complexity of soybean root-associated microbial communities. The symbiotic network analysis of bacterial community (A) and fungal community (B) at OTU level. The cooccurrence network analysis of three treatments group (C), four sampling compartments group (D) and three soybean growing stages group (E) of bacterial community at phylum level. The cooccurrence network analysis of three treatments group (F), four sampling compartments group (G) and three soybean growing stages group (H) of fungal community at phylum level. Treatment’s details were as in Supplementary Figure 2.


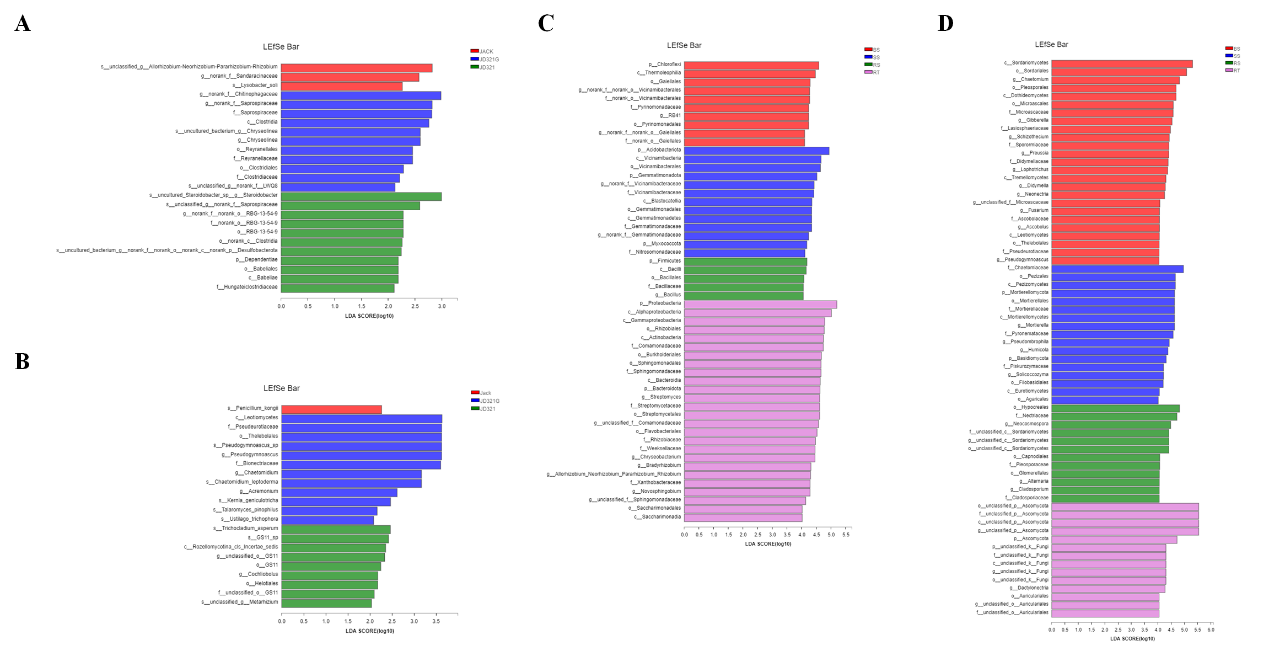


**Supplementary Figure 12** LefSe analysis of soybean root-associated microbial communities. The LefSe analysis of three treatments group of bacterial community (A) and fungal community (B) with a significant LDA threshold value of > 2. The LefSe analysis of four sampling compartments group of bacterial community (A) and fungal community (B) with a significant LDA threshold value of > 4. See treatment details in Supplementary Figure 2.
